# Extramedullary disease in multiple myeloma: a systematic literature review

**DOI:** 10.1038/s41408-022-00643-3

**Published:** 2022-03-21

**Authors:** Joan Bladé, Meral Beksac, Jo Caers, Artur Jurczyszyn, Marie von Lilienfeld-Toal, Philippe Moreau, Leo Rasche, Laura Rosiñol, Saad Z. Usmani, Elena Zamagni, Paul Richardson

**Affiliations:** 1 Department of Hematology, Hospital Clínic, IDIBAPS, University of Barcelona, Barcelona, Spain; 2 Department of Hematology, Ankara University School of Medicine, Ankara, Turkey; 3 Department of Hematology, CHU de Liège, Liège, Belgium; 4 Plasma Cell Dyscrasia Center, Department of Hematology, Jagiellonian University Medical College, Cracow, Poland; 5 Klinik für Innere Medizin II, Abteilung für Hämatologie und Internistische Onkologie, Universitätsklinikum Jena, Jena, Germany; 6 University Hospital Hotel-Dieu, Nantes, France; 7 Department of Internal Medicine II, University Hospital of Würzburg, Würzburg, Germany; 8 Department of Hematologic Oncology and Blood Disorders, Levine Cancer Institute/Atrium Health, Charlotte, NC USA; 9 IRCCS Azienda Ospedaliero-Universitaria di Bologna, Istituto di Ematologia ‘Seràgnoli’ and Dipartimento di Medicina Specialistica, Diagnostica e Sperimentale, Università di Bologna, Bologna, Italy; 10 Jerome Lipper Multiple Myeloma Center, Dana-Farber Cancer Institute, Harvard Medical School, Boston, MA USA

**Keywords:** Myeloma, Myeloma

## Abstract

Extramedullary involvement (or extramedullary disease, EMD) represents an aggressive form of multiple myeloma (MM), characterized by the ability of a clone and/or subclone to thrive and grow independent of the bone marrow microenvironment. Several different definitions of EMD have been used in the published literature. We advocate that true EMD is restricted to soft-tissue plasmacytomas that arise due to hematogenous spread and have no contact with bony structures. Typical sites of EMD vary according to the phase of MM. At diagnosis, EMD is typically found in skin and soft tissues; at relapse, typical sites involved include liver, kidneys, lymph nodes, central nervous system (CNS), breast, pleura, and pericardium. The reported incidence of EMD varies considerably, and differences in diagnostic approach between studies are likely to contribute to this variability. In patients with newly diagnosed MM, the reported incidence ranges from 0.5% to 4.8%, while in relapsed/refractory MM the reported incidence is 3.4 to 14%. Available data demonstrate that the prognosis is poor, and considerably worse than for MM without soft-tissue plasmacytomas. Among patients with plasmacytomas, those with EMD have poorer outcomes than those with paraskeletal involvement. CNS involvement is rare, but prognosis is even more dismal than for EMD in other locations, particularly if there is leptomeningeal involvement. Available data on treatment outcomes for EMD are derived almost entirely from retrospective studies. Some agents and combinations have shown a degree of efficacy but, as would be expected, this is less than in MM patients with no extramedullary involvement. The paucity of prospective studies makes it difficult to justify strong recommendations for any treatment approach. Prospective data from patients with clearly defined EMD are important for the optimal evaluation of treatment outcomes.

## Introduction

Multiple myeloma (MM) is a mature B-cell neoplasm defined by the presence of ≥10% of clonal plasma cells (PCs) in the bone marrow (or plasmacytoma confirmed by biopsy) and by evidence of end-organ damage (hypercalcemia, renal insufficiency, anemia, bone lesions) caused by the PC disorder [1]. While the PC proliferation is restricted to bone marrow in most patients with MM, a subset develops soft-tissue plasmacytomas, whereby clonal PCs escape and are found outside the bone marrow.

The presence of soft-tissue plasmacytomas represents an aggressive form of MM, characterized by the ability of a clone and/or subclone to thrive and grow independent of the bone marrow microenvironment. This is linked to high-risk genetic features, increased proliferation, evasion of apoptosis, and resistance to therapies [2, 3]. There are three principal ways that soft-tissue plasmacytomas can develop in patients with MM: (a) direct growth from skeletal tumors following cortical bone disruption; (b) growth in organs or soft tissue following hematogenous spread without contact with bony structures; and (c) rarely, growth triggered by invasive procedures [4–6].

It is important to better understand soft-tissue plasmacytomas in order to successfully direct research that will improve outcomes for affected patients. This task is complicated by the fact that several different definitions of extramedullary involvement in MM, or extramedullary disease (EMD), have been used in the published literature. A 2013 study proposed that it should comprise only of purely extramedullary plasmacytomas, and exclude bone-related plasmacytomas arising from adjacent bone marrow [7]. Other authors have contended that bone-related (or paraskeletal) plasmacytomas should be included under the definition of EMD, albeit as a distinct sub-type from extramedullary plasmacytomas [8, 9]. The former definition and distinction has been endorsed by a recent expert consensus review [4]. Solitary plasmacytomas (SP) should not be considered as EMD, since they occur in the absence of an MM diagnosis [9, 10]. Plasma cell leukemia (PCL) is also typically excluded from the definition of EMD, on the basis that it is a well-characterized pathologic entity with distinct prognostic implications and treatment recommendations [9, 11–13]. Definitions of these different clinical entities are shown in Table 1.

**Table 1:** Definitions of plasma cell neoplasms.

Plasma cell neoplasm	Definition
Extramedullary disease	An aggressive form of multiple myeloma characterized by the presence of soft-tissue plasmacytomas that result from hematogenous spread
Paraskeletal plasmacytoma	A form of multiple myeloma characterized by the presence of soft-tissue plasmacytomas that occur due to direct growth from skeletal tumors following cortical bone disruption
Solitary plasmacytoma	A single mass of clonal plasma cells (bone or extramedullary) with no or minimal BM plasmacytosis and with no other symptoms than those derived from the primary lesion
Plasma cell leukemia	A rare and aggressive variant of myeloma characterized by the presence of circulating plasma cells; diagnosis is based upon the percentage (≥20%) and absolute number (≥2 × 10^9^/L) of plasma cells in peripheral blood

Several genetic features have been linked to extramedullary involvement in MM. These include 17p deletion [14, 15], nuclear expression of p53 [16], a higher incidence of t(4;14) [17], and p53 deletion [18]. Other possible risk factors include loss of CD56 expression [19], MAFB overexpression [20], and MYC overexpression [21]. Treatment with novel agents has also been suggested as a risk factor for the emergence of EMD, although the data to support this are equivocal [4].

This review is based on a systematic literature search, and will focus on hematogenous EMD, including that with central nervous system (CNS) involvement. Areas covered are incidence, sites of occurrence, prognosis, and treatment outcomes.

## Methods

The PubMed database was searched for publications of interest from January 1, 2010 to 31 December, 2020, using the following search terms: [Extramedullary AND (myeloma OR plasmacytoma)] OR [myeloma AND (bone-related OR soft tissue OR extraosseous)] OR [central nervous system AND (myeloma OR plasmacytoma OR extramedullary)]. Supplemental literature searches (for treatment outcomes) of proceedings from the following relevant conferences that took place in 2019 and 2020 were also conducted: European Haematology Association 2019 and 2020; American Society of Clinical Oncology 2019 and 2020; American Society of Hematology 2019 and 2020; International Myeloma Working Group 2019.

Following a check for duplicates, retrieved records (titles and abstracts) were screened to exclude those clearly not relevant for inclusion. At this stage, records were excluded on the basis of the following criteria: non-English language, review articles, preclinical studies, or individual case studies/case series involving fewer than 10 patients. Full-text articles for records deemed to be possibly relevant were then obtained and reviewed for inclusion in the qualitative synthesis (given the nature of the data it was not possible or appropriate to conduct a quantitative analysis). To be included, an article needed to clearly report data for hematogenous EMD and to clearly define the phase of MM (NDMM or RRMM); it also needed to include data for one or more of the areas of interest (sites of occurrence, incidence, prognosis, treatment outcomes, CNS EMD).

## Results

**Fig. 1:**
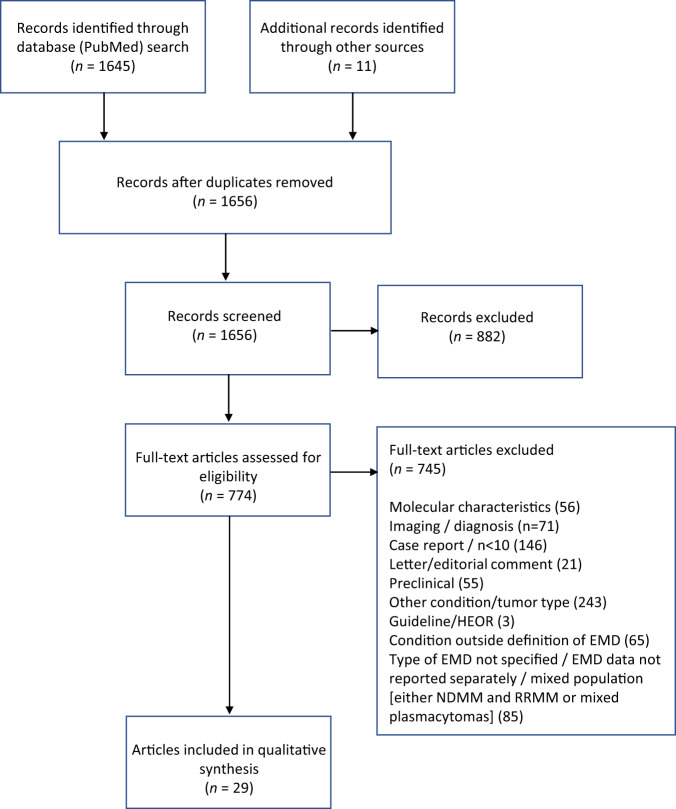
PRISMA flow diagram for qualitative synthesis.

A total of 29 articles were included in the qualitative synthesis. The PRISMA flow diagram is shown in Fig. 1.

### Incidence

In some patients, EMD can be identified as visible or palpable masses; however, imaging techniques are usually needed. Both paraskeletal plasmacytomas and EMD can be found at diagnosis of MM (sometimes referred to as primary), or at relapse (secondary) [20, 22, 23]. The reported incidence varies considerably across studies, most likely due to differences in the definitions applied and the methods of evaluation. Magnetic resonance (MRI) is the best imaging approach for spinal and central nervous system (CNS) involvement. However, functional whole-body techniques should ideally be used to detect EMD. A consensus statement by the International Myeloma Working Group (IMWG) specifically recommends ^18^F-FDG PET/CT for this purpose [24]. Hopefully, the previous lack of standardization is solved with the recent proposal of PET/CT standardization score according to the Deauville criteria based on the analysis of two prospective studies [25]. The reported incidence of EMD at diagnosis before the PET/CT era was low, ranging from 1.7 to 4.5% [4]. With the use of PET/CT, the incidence of EMD at diagnosis is between 6 and 10% [23, 24, 26, 27], which should help to stimulate sub-studies for EMD in prospective clinical trials (such as the CASSIOPET study of the CASSIOPEIA trial). Finally, PET/CT should be performed in clinical practice for all patients with a suspicion of EMD, such as those with high LDH serum levels or revised stage III [4].

A number of studies have reported incidence data specifically for EMD (Table 2). A meta-analysis of eight clinical trials in patients with NDMM, involving 2332 patients, found an overall incidence of soft-tissue plasmacytomas of 11.4%, and an incidence of EMD of 0.5% [26]. The authors acknowledge that their reported incidence of EMD is likely to be an underestimation due to the imaging techniques used in the study. A study of 3744 patients with NDMM reported an overall incidence of soft-tissue plasmacytomas of 18.2%, with paraskeletal involvement in 14.5% and extramedullary involvement in 3.7% [28]. Deng et al. [18] assessed EMD in 834 consecutive MM patients from a single center in China and found incidence rates of 4.8% and 3.4% in patients with NDMM and RRMM, respectively. Another single-center study in Boston, USA, found EMP in 55/663 consecutive patients (8.3%) undergoing stem-cell transplantation [29]. Among these 55 patients, EMD was present at diagnosis in eight, at relapse in 42, and at both diagnosis and relapse in five. An analysis of 226 patients with RRMM found soft-tissue plasmacytomas in 24% of patients; this was adjacent to bone (paraskeletal) in 10% and not adjacent in 14% [23]. Rasche et al. [21] screened their myeloma registry for patients who developed EMD relapse, and reported an incidence rate of 6.7% (24/357 patients). In a Mayo Clinic including 174 patients with RRMM enrolled in a phase 2 clinical trial [30], 16 patients (9.2%) were found to have EMD (3 patients [1.7%] at the time of diagnosis, 13 [7.5%] during the course of treatment).

**Table 2:** Incidence rates of EMD in studies of patients with MM.

Reference	No. of patients	Time period covered	Diagnostic approach	Incidence
Montefusco et al. [26]	2322	2010–2018 (across all studies)	Skeletal survey, MRI, or CT; and/or physical examination	NDMM: 0.5%
Gagelmann et al. [28]	3744	2005–2014	NR	NDMM: 3.7%
Deng et al. [18]	834	1993–2013	X-ray, US, CT, physical examination; histologically confirmed where possible	NDMM: 4.8% RRMM: 3.4%
Weinstock et al. [29]	663	2005–2011	Pathological or radiological evidence of EMD at any time following the initial diagnosis of MM	RRMM: 8.3%
Pour et al. [23]	226	2005–2008	US, CT, or MRI	RRMM: 14%
Rasche et al. [21]	357	2007–2010	Cytology, biopsy of clinical/radiological lesions	RRMM: 6.7%
Short et al. [30]	174	2007–2011	PET/CT, MRI	NDMM: 1.7% RRMM: 7.5%

*CT* computed tomography, *MRI* magnetic resonance imaging, *NR* not reported, *PET* positron emission tomography, *US* ultrasound.

### Typical sites of occurrence

Plasmacytomas can be found in virtually any area of any tissue in the body. A recent real-world retrospective study of 226 MM patients presenting with plasmacytomas included 176 patients with EMD [31]. The most common sites of EMD occurrence were skin/muscle (24%), pleura (12%), lymph nodes (10%), liver (9%), and CNS (6%). Different sites appear to be involved in EMD identified at MM diagnosis and at relapse, with skin the most commonly involved tissue in NDMM [2, 20, 28, 29]. In RRMM, sites involved include liver, kidneys, lymph nodes, CNS, breast, pleura, and pericardium [2, 28, 29].

### Prognosis

A retrospective study of transplant-eligible patients with MM compared survival outcomes in patients who had EMD (*n* = 17), bone-related plasmacytomas (*n* = 22), and no plasmacytoma involvement (*n* = 141) at diagnosis [32]. Five-year overall survival (OS) was 63% in patients who had bone-related plasmacytomas, 63% in patients who had EMD, and 80% in patients without plasmacytomas at diagnosis (*p* = 0.02). Five-year disease-free survival was 47% in patients who had bone-related plasmacytomas, 35% in patients with EMD, and 54% in MM patients who did not have plasmacytomas (*p* = 0.15). Moreau et al. [33] found that absence of EMD at diagnosis was an independent prognostic factor for longer progression-free survival (PFS; *p* < 0.001) and OS (*p* = 0.004). By multivariate analysis, the hazard ratios (HRs) for PFS and OS were 3.394 (95% CI 2.055–5.606) and 3.894 (95% CI 1.540–9.851), respectively. Another study assessed predictors of inferior clinical outcome in patients (*n* = 51) with standard-risk MM [34]; on univariate analysis, EMD was associated with significantly shorter OS (HR 3.05; 95% CI 0.57–16.29; *p* = 0.02).

### Treatment outcomes

#### EMD in patients with NDMM

Currently available data for treatment outcomes in patients with NDMM and EMD are summarized in Table 3.

**Table 3:** Treatment outcomes in patients with NDMM and EMD.

Reference Study type	Total number of patients/ number with EMD	Time period covered	Treatments	PFS	OS
Montefusco et al. [26] Meta-analysis of 8 trials	267 EMD, *n* = 12	2010–2018 (across all studies)	IMiD-based therapy (*n* = 166), PI-based (*n* = 66), IMiD+ PI-based (*n* = 35)	Median (95% CI): 26.1 months (8.0–NR)	2-year: 35% Median (95% CI): 70.1 months (16.9–NR)
Batsukh et al. [35] Retrospective	64 EMD, *n* = 22	2009–2016	Bortezomib/dexamethasone (*n* = 7) Thalidomide/dexamethasone (*n* = 23) Bortezomib/thalidomide/dexamethasone (*n* = 11) Bortezomib/melphalan/prednisone (*n* = 23) Lenalidomide/dexamethasone (*n* = 1) Mlphalan/prednisolone or dexamethasone (*n* = 6) ASCT (*n* = 28)	Median (95% CI): 16.0 months (5.8–26.2)	Median (95% CI): 27.8 months (5.8–26.5)
Beksac et al. [31] Retrospective	130 EMD, *n* = 92	2010–2017	Median two lines of treatment and ASCT (44%)	Median (95% CI): 38.9 months (23.6–54.2)	Median (95% CI): 46.5 months (25.5–67.5)
Gagelmann et al. [36] EBMT registry analysis	488 EMD, *n* = 87	2003–2014	Induction with bortezomib (*n* = 355) vs non-bortezomib (*n* = 133) Transplants: autologous (*n* = 373) or tandem autologous (*n* = 84) or autologous-allogeneic transplant (*n* = 31)	3-year (range): 39% (27–52)	3-year (range): 60% (48–73)
Gagelmann et al. [28] EBMT registry analysis	682 EMD, *n* = 139	2005–2014	Upfront single ASCT within 12 months of diagnosis or a tandem ASCT within 6 months from first ASCT as first-line therapy	3-year (range): 39.9% (30.3–49.5)	3-year (range): 58.0% (48.1–67.9)

*ASCT* autologous stem-cell transplantation, *EBMT* European Society for Blood and Marrow Transplantation, *EMD* extramedullary disease, *IMiD* immunomodulatory drugs, *OS* overall survival, *PFS* progression-free survival, *PI* proteasome inhibitor.

The recent meta-analysis of eight trials of IMiD- or PI-based therapy in patients (*n* = 2332) with NDMM [26] included 267 patients with soft-tissue plasmacytomas (paraskeletal, *n* = 243; EMD, *n* = 12; not classified, *n* = 12). Median PFS was 26.1 months and 25.2 months in patients with EMD and patients without plasmacytomas, respectively; median OS was 70.1 months (EMD) and 79.9 months (no plasmacytomas). Batsukh et al. [35] retrospectively assessed outcomes in 64 NDMM patients with soft-tissue plasmacytomas who were treated with various regimens containing novel agents. Median PFS was similar (approximately 16 months) for patients with bone-related plasmacytomas and EMD, while median OS was significantly shorter (27.8 vs 54.2 months, *p* = 0.033) in the EMD group (mainly due to shorter PFS after first relapse).

Beksac et al. [31] conducted a multicenter, multinational retrospective study of 226 MM patients with plasmacytoma involvement; 176 had EMD (*n* = 92 at MM diagnosis, *n* = 84 at relapse) and 50 had paraskeletal plasmacytomas (*n* = 38 at MM diagnosis, *n* = 12 at relapse). The entire group received a median two lines of treatment and autologous SCT (44%) following their plasmacytoma diagnosis. PFS and OS were 38.9 months and 46.5 months for EMD at diagnosis and 51.7 months (*p* = 0.034) and not reached (*p* = 0.002) for paraskeletal plasmacytomas.

Gagelmann et al. [36] analyzed clinical and cytogenetic data from 488 patients with NDMM and soft-tissue plasmacytomas (paraskeletal, *n* = 374; EMD, *n* = 87; both, *n* = 27) who underwent SCT. High-risk cytogenetics were identified in 41% of the patients and found more frequently in those with EMD. Outcomes following single autologous SCT were significantly worsened in the presence of high-risk cytogenetic abnormalities, whereas a tandem autologous transplant strategy was shown to offset the poor prognosis. An analysis of 3744 patients with NDMM found no difference in 3-year PFS following first-line autologous SCT between those with single-site plasmacytomas (any location) and patients without plasmacytomas [28]. However, single EMD involvement was associated with worse 3-year OS compared with no plasmacytomas, which worsened still further when multiple sites of organs were involved. Note that there is some overlap in the patients included in these two studies [28, 36].

#### EMD in patients with RRMM

A summary of currently available data for treatment outcomes in patients with RRMM and EMD is provided in Table 4.

**Table 4:** Treatment outcomes in patients with RRMM and EMD.

Reference Study type	Total number of patients/ number with EMD	Time period covered	Treatments	PFS	OS	Other
**Novel agents (various) and/or SCT**						
Avivi et al. [37] Retrospective	127 (all EMD)	2010–2018	First treatment included PIs (50%), IMiDs (39%), monoclonal antibodies (10%), and chemotherapy (53%)	–	–	57% ORR ( ≥ PR) across all treatments
Rasche et al. [21] Single-center registry	24 (all EMD)	2007–2010	Radiotherapy (*n* = 16) ASCT + intense dose chemotherapy (*n* = 6) Bortezomib (*n* = 16) Lenalidomide (*n* = 12) Thalidomide (*n* = 8)	Median (95% CI): 2 months (0.08–3.92)	Median (95% CI): 7 months (3.56–10.43)	–
Beksac et al. [31, 38] Retrospective	96 EMD, *n* = 84	2010–2017	Median two lines of treatment and ASCT (44%)	Median (95% CI): 9.1 months (11.6–15.6)	Median (95% CI): 11.4 months (0.6–16.2)	Complete remission rate: 9% (vs 54.5% in patients with paraskeletal, *p* < 0.001)
**IMiDs**						
Short et al. [30] Phase 2 trial	16 (all EMD)	2007–2010	Pomalidomide + low-dose dexamethasone	–	Median: 16 months	–
**PIs**						
Zhou et al. [39] Retrospective	45 EMD, *n* = 25		Carfilzomib + dexamethasone-based	EMD significantly inferior PFS vs paraskeletal (*p* = 0.004)	EMD significantly inferior OS vs paraskeletal (*p* = 0.04)	–
Papanikolaou et al. [40] Retrospective	28 (all EMD)	1998–2011	At relapse:Bortezomib-containing regimens (32%) Platinum-containing (21%) Lenalidomide (21%) VAD (8%)	–	Median following relapse: 5 months Median from MM diagnosis: 38 months	–
**Chemotherapy/radiotherapy**						
Rasche et al. [41] Retrospective	11 (all EMD)	2007–2012	Dexa-BEAM (including dexamethasone, carmustine, cytarabine, etoposide, and melphalan)	Median: 4 months	–	Objective response (≥PR) achieved in 6/11 patients
**Recently approved and investigational agents**						
Richardson et al. [42] Phase 2	55 EMD, *n* = 27	Patients enrolled between Dec 2016 and Oct 2019	Melflufen + dexamethasone	–	–	ORR: Non-EMD, 32% Paraskeletal, 25% EMD, 22%
Wang et al. [43] Single-center	57 EMD, *n* = 17	2016–2018	LCAR-B38M	Median: 8.1 months (vs 25 months in non-EMD; *p* < 0.001)	Median: 13.9 months (vs NR in non-EMD; *p* = 0.0019)	82% ORR (≥ PR) vs 90% for non-EMD

*ASCT* autologous stem cell transplantation, *EMD* extramedullary disease, *MM* multiple myeloma, *NC* not calculable, *NR* not reached, *ORR* overall response rate, *OS* overall survival, *PFS* progression-free survival, *PR* partial response, *VAD* vincristine/adriamycin/dexamethasone.

In a retrospective study of 127 consecutive patients with hematogenous EM relapse [37], first treatments for EMD included PIs (50%), IMiDs (39%), monoclonal antibodies (10%), and chemotherapy (53%). ORR (≥ partial response) was 57% across all treatments and IMiDs were associated with higher ORR compared with PIs (HR 2.2, 95% CI 1.02–4.7; *p* = 0.04). A single-center analysis of 24 patients with EMD on relapse reported very poor prognosis (median PFS of 2 months and median OS of 7 months) despite treatment with novel agents in 22 of the 24 patients [21].

In the multinational retrospective study conducted by Beksac et al. [31, 38], patients with EMD at relapse had a PFS and OS of 9.1 months and 11.4 months, respectively. The complete remission rate was significantly lower than the rate in patients with bone-related plasmacytomas (9% vs 54.4%, *p* = 0.001).

Short et al. [30] studied the detailed medical records of 174 consecutive patients with RRMM who enrolled in a phase 2 clinical trial of pomalidomide plus low-dose dexamethasone. Sixteen patients had EMD, and these patients had significantly worse OS from trial entry compared with non-EMD patients (median 16 months vs not reached; *p* = 0.002).

Zhou et al. [39] retrospectively assessed carfilzomib-containing therapies in 45 patients with RRMM and invovlement of plasmacytomas. PFS and OS are not reported separately for bone-related plasmacytomas and EMD, although EMD without adjacency to bone was associated with a significantly shorter PFS (*p* = 0.004) and OS (*p* = 0.04) compared with paraosseous lesions. In a study of 303 patients with MM, including 28 cases of EMD relapse, prior treatment with bortezomib was associated with a decreased hazard of EMD relapse (*p* = 0.041) [40]. Median OS from MM diagnosis was significantly shorter in the group with EMD relapse than in the non-EMD group (38 months vs 59 months; *p* = 0.006).

Rasche et al. [41] retrospectively analyzed the polychemotherapy regimen, Dexa-BEAM, in 18 patients with advanced MM (11 had EMD). Objective response (≥ partial response) to Dexa-BEAM was achieved in more than half (6/11) of the patients; subsequent high-dose consolidation strategy with autologous or allogeneic SCT improved upon the depth of remission in two-thirds of EMD patients (4/6) with ongoing remissions in three patients.

A phase 2 study (HORIZON) has assessed melflufen (plus dexamethasone) in patients with heavily pretreated RRMM, and a subgroup analysis of patients with involvement of plasmacytomas has been reported [42]. The study enrolled 157 patients, including 55 with plasmacytomas (bone-related, *n* = 28; EMD, *n* = 27). The ORR was 32% in the group without plasmacytoma involvement, 25% in the group with bone-related plasmacytomas, and 22% in the EMD group.

The CAR-T therapy LCAR-B38M has been assessed in a single-center study involving 57 patients (17 with EMD) [43]. At a median follow-up of 25.1 months, median PFS was 8.1 months in the EMD group and 25 months in those without EMD (*p* < 0.001); median OS was 13.9 months in the EMD group and not reached in those without EMD (*p* = 0.0019). Published trials of other CAR-T therapies have reported responses in patients with soft-tissue plasmacytomas, but do not report data specifically for patients with EMD [44, 45]. Very recently, data have been presented from a phase 1b/2 study of ciltacabtagene autoleucel [46]; 13 patients with EMD were included, and the authors report similar response rates in the EMD subgroup to those of the overall study population (ORR 94.7%).

Deng et al. [47] have recently reported on the safety and efficacy of humanized anti-B-cell maturation antigen (BCMA) CAR-T therapy in 7 patients with EMD compared with 13 with no EMD involvement. Cytokine release syndrome (CRS) and immune effector cell-associated neurotoxic syndrome (ICANS) were higher in patients with EMD, most likely related to the higher tumor burden in this population. While the response rate was similar in both groups, the 1-year PFS and OS rates were significantly shorter in patients with EMD. The unsatisfactory long-term efficacy of anti-BCMA CAR-T therapy in EMD, also highlighted by Wang et al. [43], is worrisome and needs to be further explored in forthcoming trials.

### CNS involvement

Available data for treatment outcomes in patients with CNS soft-tissue involvement are summarized in Table 5. In a large (*N* = 172), retrospective, multi-institutional study, overall median OS after onset of CNS involvement was 6.7 months [48]. Median OS was 2 months for those untreated and 8 months for patients who received treatment for CNS disease. Another study of 16 patients treated with different combinations of systemic therapy, intrathecal chemotherapy, and radiotherapy highlighted a dismal outcome for patients with leptomeningeal involvement (median OS of 82 days) [49].

**Table 5:** Treatment outcomes in studies of patients with CNS involvement.

Reference Study type	No. of patients	Time period covered	Treatments	PFS	OS
Bommer et al. [49] Retrospective	16 (all LMM)	2005–2016	Intrathecal chemotherapy, radiotherapy	–	Median OS: 82 days (after LMM diagnosis)
Jurczyszyn et al. [48] Retrospective	172 (38 at initial MM diagnosis, 134 at relapse/progression)	1995–2014	Systemic therapy (*n* = 117) Radiotherapy (*n* = 56) Intrathecal therapy (*n* = 49) Steroids only (*n* = 5) Mass resection (*n* = 1) SCT (*n* = 32)	–	Median OS: 6.7 months (all patients) 2 months (untreated patients) 8 months (treated patients)
Paludo et al. [50] Retrospective	29 (7 at initial diagnosis of MM, 22 at relapse)	1998–2014	Radiation therapy (*n* = 22) + adjuvant intrathecal chemotherapy (*n* = 6) Intrathecal + systemic therapy (*n* = 1) Systemic therapy (*n* = 2) Novel agents, including bortezomib (28%), thalidomide (14%), lenalidomide (10%), and pomalidomide (3%), administered after CNS involvement. ASCT after the diagnosis of CNS disease (24%)		Median OS (95% CI): CNS involvement, 40 months (24–56) Control (no CNS involvement), 93 months (67–129) Patients with ASCT after CNS involvement, 19 months (10–67)
Katodritou et al. [51] Retrospective	31	2000–2013	Bortezomib-based (*n* = 12) IMiD-based (*n* = 5) Chemotherapy alone (*n* = 8) Intrathecal infusions (*n* = 3) Additional radiotherapy (*n* = 9)	Median (95% CI) CNS involvement, 16 months (2–30.6) Control (no CNS involvement), 36 months (12–60) *p* = 0.004	Median (95% CI) CNS involvement, 47 months (32–62) Control (no CNS involvement), 84 months (31–137) *p* = 0.01
Abdallah et al. [52] Retrospective	35	1996–2012	Chemotherapy, including intrathecal (*n* = 28) Intrathecal alone (*n* = 3)	–	Median (range): 4 months (1–13)
Chen et al. [53] Retrospective	37	1999–2010	Intrathecal chemotherapy (81%) Cranial and/or spinal irradiation (78%) IMiDs (51%) Cisplatin-based (27%) Bortezomib (19%) Alkylators (11%) Dexamethasone alone (8%) ASCT (5%)	Median (95% CI) after CNS involvement: 3.1 months (2.0–6.0)	Median (95% CI) after CNS involvement: 4.6 months (2.8–6.7)
Lee et al. [54] Retrospective	17	2000–2011	Systemic pharmacotherapy Intrathecal chemotherapy and/or radiotherapy	–	Median (range) after CNS involvement: 4 months (1–23)
Gozzetti et al. [55]	12	2000–2010	Systemic treatment Systemic treatment + radiotherapy Systemic + radiotherapy + intrathecal Radiotherapy + intrathecal Intrathecal (note: specific treatments received by patients with CNS-MM not specified)	–	Median (range): 6 months (1–23)

*ASCT* autologous stem cell transplantation, *CNS* central nervous system, *IMiD* immunomodulatory drug, *LMM* leptomeningeal myelomatosis, *MM* multiple myeloma.

Paludo et al. [50] compared a cohort of MM patients with CNS involvement (*n* = 29) with a control population of patients without CNS involvement. OS from diagnosis of MM was shorter in the CNS-MM group than in the control group (median 40 months vs 93 months); OS from detection of CNS involvement was 3.4 months. In patients who underwent ASCT after CNS involvement (*n* = 7), median OS was 19 months (95% CI 10–67 months) from the detection of CNS involvement.

Katoditrou et al. [51] retrospectively reviewed medical records of 31 patients with CNS-MM treated in centers from Greece. Both PFS and OS were significantly shorter in the patients treated for CNS-MM (*n* = 29) compared with the control group (MM with no CNS involvement), and treatment with novel agents did not confer a survival advantage. Another study retrospectively reviewed patients with CNS-MM (*n* = 35) identified from the University of Arkansas MM database. [52] Treatment comprised mainly systemic and/or intrathecal chemotherapy, and median OS was 4 months.

Yet another retrospective study, from a single center in Canada, identified 37 patients with CNS-MM [53]. Median OS was only 4.6 months, although nine patients had prolonged survival (median 17.1 months); these longer-term survivors were treated with radiotherapy, intrathecal chemotherapy, and IMiD-based regimens. Lee et al. [54] retrospectively analyzed 17 patients with CNS-MM, reporting a median OS of 4 months from time of CNS involvement. OS was significantly better in patients who received intrathecal chemotherapy than those who did not (20 months vs 2 months, respectively; *p* < 0.02). Another retrospective survey of 50 patients with intracranial involvement in MM included 12 patients with CNS-MM [55]; within the subgroup of patients with CNS involvement, the median survival was 6 months.

## Discussion

The presence of soft-tissue plasmacytomas represents an aggressive form of MM, which can be found at the time of MM diagnosis or at relapse. Several different definitions of extramedullary involvement in MM or EMD have been proposed in the literature. We and others advocate that true EMD is restricted to plasmacytomas that arise due to hematogenous spread and have no contact with bony structures. Typical sites of EMD may vary according to the stage of MM. At diagnosis, EMD is typically found in skin; at relapse, typical sites involved include the liver, kidneys, lymph nodes, breast, pleura and pericardium, and the CNS.

In addition to the variation in definition, the published literature on soft-tissue plasmacytomas is difficult to navigate for several reasons: data may be reported for ‘mixed’ populations of patients with NDMM and RRMM; data may be reported for bone-related plasmacytomas and EMD combined; or a clear definition is lacking the type of plasmacytoma being studied. By including, as far as possible, only studies that clearly define the phase of MM and clearly specify EMD, this review differs from much of the previously published literature in this area.

The reported incidence of EMD varies considerably, and differences in diagnostic approach between studies are likely to contribute to this variability. In patients with NDMM, the reported incidence ranges from 0.5% to 4.8%, while in RRMM the reported incidence is 3.4–14%. Available data demonstrate that the prognosis is poor, and considerably worse than for MM without EMD. For patients with soft-tissue plasmacytomas, those with EMD typically have poorer outcomes than those with paraskeletal involvement. CNS involvement is rare, but prognosis is even more dismal than for EMD in other locations, particularly if there is leptomeningeal involvement.

The outcome of patients with EMD should be reported as a predefined subgroup in clinical trials. In this regard, the prospective IMAJEM study from the French group showed the prognostic impact of the presence of EMD [33]. In the CASSIOPEIA study, from the same group comparing bortezomib/thalidomide and dexamethasone with or without daratumumab as pretransplant induction regimen, a PET/CT substudy (CASSIOPET) is ongoing, which aims to investigate the prognostic impact and response to therapy, including MRD assessments, in patients with EMD [27]. Hopefully, the design of future trials will investigate the prognostic impact and treatment efficacy in patients with EMD.

Available data on treatment outcomes for EMD are almost entirely derived from retrospective studies. Some agents and combinations have shown a degree of efficacy but, as would be expected based on known prognoses, this is typically less than in MM patients with no extramedullary involvement. The paucity of prospective studies makes it difficult to justify strong recommendations for any treatment approach. The recent expert consensus review provided some possible treatment approaches for consideration [4]. For upfront treatment of EMD in transplant-ineligible patients, the addition of daratumumab to VMP or RVD was suggested. In transplant-eligible patients, intensive anti-myeloma/anti-lymphoma regimens (e.g. VTD/ or VRD/PACE combined with SCT) are proposed as a theoretical option [4]. Suggested treatments at relapse are also based on lymphoma-like regimens such as PACE, DCEP, or Dexa-BEAM, although duration of response is typically ≤4 months. Beyond this, novel-agent combinations (e.g. carfilzomib-, selinexor-, or isatuximab-based) or newer/investigational agents (e.g. CAR-T, BiTEs, melflufen) may be considered [4]. For CNS involvement, some combination of IMiD-based systemic therapy, intrathecal and radiation therapy appears to provide the best treatment outcome.

Current response criteria, including MRD assessment, should be applied in patients with EMD. As per the recent consensus on extramedullary involvement in MM, the first assessment of EMD identified by PET/CT (considering size and metabolic uptake) and/or MRI should be done at three months after treatment initiation and at physician discretion thereafter [4]. It is recommended that baseline and follow-up assessments should use the same imaging technique in order to minimize the inter-technique variability. To declare complete remission (CR), all evidence of EMD must have disappeared according to the standardization for metabolic CR definition recently proposed [25], as well as the disappearance of the serum and urine M-protein by immunofixation [56]. In addition, a bone marrow negative for MRD by flow cytometry (FC) will define not only CR, but also MRD negativity (so-called double negative at EM involvement PET/CT and at bone marrow FC) [27, 33]. There is an expectation that liquid biopsy with MRD assessment in peripheral blood may help to further ensure disease eradication in all compartments; however, data on this remains limited.

Prospective data from patients with clearly defined EMD are important for the optimal evaluation of treatment outcomes. Conducting trials that are adequately powered to assess outcomes is a challenge in this uncommon group of patients. In such cases, the inclusion of these patients in trials to allow subgroup assessment of treatment effects with a priori hypotheses may provide the best attainable evidence. There are signs from trials of the next wave of MM treatments that patients with EMD are at last being studied in greater detail, and this is a trend that should be encouraged. Widespread adoption of specific response criteria based on both morphological and functional evaluation, such as those proposed recently by Zamagni et al. [25], will also be important for understanding and comparing the impact of different treatments on EMD.
